# The NewGait Rehabilitative Device Corrects Gait Deviations in Individuals With Foot Drop

**DOI:** 10.1155/2024/2751643

**Published:** 2024-09-11

**Authors:** Ksenia I. Ustinova, Joseph E. Langenderfer

**Affiliations:** ^1^ Department of Physical Therapy Central Michigan University, Mount Pleasant, Michigan, USA; ^2^ School of Engineering and Technology Central Michigan University, Mount Pleasant, Michigan, USA

**Keywords:** assistive devices, coordination, gait, orthoses

## Abstract

The purpose of this quasiexperimental study was to test the effects of wearing the NewGait rehabilitative device on walking abilities in individuals with foot drop. The study involved 16 participants with foot drops caused by stroke (11 participants), multiple sclerosis (one participant), and peripheral neuropathies (four individuals). During a single testing session, participants walked 12 m at their self-selected speed in four experimental conditions: walking without any orthotic device; walking while wearing a regular plastic posterior leaf ankle foot orthosis (AFO); walking with the NewGait device assisting ankle dorsiflexion only; and walking with the NewGait device assisting the hip, knee, and ankle joint motions. Body motions during walking were recorded using a 3D system for motion analysis and analyzed with a set of spatiotemporal and kinematic parameters and a gait decomposition index. The gait decomposition index indicated sagittal interjoint coordination in the three joint pairs (hip–knee, knee–ankle, and hip–ankle) of the paretic (foot drop) leg during walking and was validated in a previous study. Overall, wearing all three orthotic devices improved the gait velocity, ankle dorsiflexion, and foot clearance compared to gait trials in which no assistive devices were used. However, wearing the AFO significantly restricted the plantarflexion range of motion and decreased interjoint coordination as measured by joint decomposition. In contrast, the NewGait device altered the ankle plantarflexion motions but also increased coordinated movement (reduced the decomposition) in most lower-extremity joint pairs and conditions. Therefore, the NewGait rehabilitative device can be considered superior to a regular AFO in correcting gait deviations caused by foot drop.

## 1. Introduction

Foot drop is a common medical term used by physical medicine clinicians to describe gait deviations due to neurologically induced weakness of muscle dorsiflexors [1]. This condition is often caused by peripheral peroneal neuropathies or disorders of the central nervous system such as stroke, multiple sclerosis, or cerebral palsy [2]. As a result, the foot is unable to clear the ground during the swing phase of gait, which increases the risk of tripping and falling. Another problem created by full or partial denervation of dorsiflexors is the inability to control the foot landing on the ground during the loading response, resulting in a prominent foot “slap” [3]. To compensate for these abnormalities, individuals may develop various strategies to make walking more energy-consuming and less effective, which negatively affects their quality of life.

Many orthotic devices have been developed to address foot dorsiflexion during gait, with plastic ankle foot orthoses (AFOs) being the most commonly used type [4, 5]. These devices assist dorsiflexion while restricting plantarflexion to a slightly dorsiflexed or neutral end range of motion. While standard AFOs provide a simple, ergonomic, affordable, and quick solution for fixing gait deviations associated with dorsiflexor weakness, these devices have their own drawbacks. Wearing plastic or carbon fiber AFOs with insoles restricts motion at the ankle joint not only in the sagittal plane but also in other orthogonal planes. This restriction can affect interjoint and synergetic leg coordination, making the overall leg motion less complex and less adaptable to the requirements of the external environment and activities of daily living tasks [6]. Therefore, there should be other orthopedic solutions to correct foot drop.

The NewGait device provides an alternative approach to the traditional use of plastic or carbon fiber material for the correction of foot drop. Instead, elastic (rubber) bands of varying resistance are attached to straps around the thighs, waist, and chest, and a waist belt. When the bands are stretched by moving the legs, external contractile forces in the bands generate torques that assist the weak dorsiflexors. The device has two main configurations. The first configuration is similar to an AFO and provides assistance to the muscles that dorsiflex and evert the foot. The second configuration is more complex and simultaneously assists the hip, knee, and ankle joints in all three planes, if necessary.

To date, the NewGait system has been tested in a series of case studies in individuals with stroke, cerebral palsy, multiple sclerosis, and sports injuries of the lower extremities [7, 8]. The device has been shown to be effective in correcting gait abnormalities in single individuals likely representative of neurological populations [8]. However, no experimental comparison of NewGait with a commonly used assistive device has been reported that can be generalized to a population of individuals with foot drop. Therefore, the purpose of the present study was to test whether wearing the NewGait rehabilitative device helps correct gait abnormalities in individuals with foot drop. A specific question in the study was whether the NewGait is comparable to a regular plastic AFO in correcting gait abnormalities.

## 2. Methods

### 2.1. Participants

A convenience sample of 16 individuals with foot drop due to lower extremity paresis caused by stroke (11 individuals), multiple sclerosis (one individual), and traumatic and compressive neuropathy (four individuals) participated in the study. The sample size was calculated based on data collected in a previous experiment where gait kinematics were measured in a small number of individuals with foot drop, both when walking with and without the NewGait device [8]. The sample size was determined to ensure a statistical power of at least 80% at a significance level of 0.05, with an effect size of 0.9 or greater. Ten of 16 individuals had paresis on the left side, or their left lower extremity was more affected than their right extremity. The average age was 61 ± 11.47 years, and the average time since disease onset was 7.9 ± 7.9 years (range from 1.5 to 30 years). Because the causes of lower extremity paresis were different in the study participants, different scales were used to evaluate the severity of their neurological condition. Gait speed was used to bring the severity of participants' conditions to a common denominator. Gait speed is recognized as one of the universal parameters of quality of life, with a speed of 1.2–1.4 m/s considered normal. A speed below 0.8 m/s is indicative of limited community ambulation [9], and below 1.0 m/s is a strong predictor of the risk of falling and requiring intervention [10]. The average speed of comfortable walking in the study participants was 0.65 ± 0.25 m/s, ranging from 0.24 to 1.13 m/s and encompassing a full range of mild to severe gait impairments. Six participants used assistive devices for ambulation (single-point or quad canes), and eight participants wore AFOs regularly.

All participants were recruited from the list of current or former patients in local inpatient and outpatient clinics and signed a consent form approved by the Institutional Review Board before participation. To be eligible for the study, participants had to have foot drop and resultant gait deviations; be able to walk independently at least 30 m, but at a speed slower than 1.2 m/s; have no pain or recent orthopedic traumas or surgeries affecting the lower extremities; and not be excluded if they needed assistance from a cane or walker. They were motivated to participate by the opportunity to explore an alternative orthotic solution that could potentially improve or at least ease their gait, as they had not previously found conventional orthotic prescriptions useful or feasible for them. A walking speed of 1.2 m/s was chosen as the cut-off speed, as this is the speed indicative of gait deviations that require intervention [11, 12], and the inability to walk at a speed of 1.2 m/s was indicative of gait impairments.

### 2.2. Equipment and Experimental Procedures

**Figure 1:**
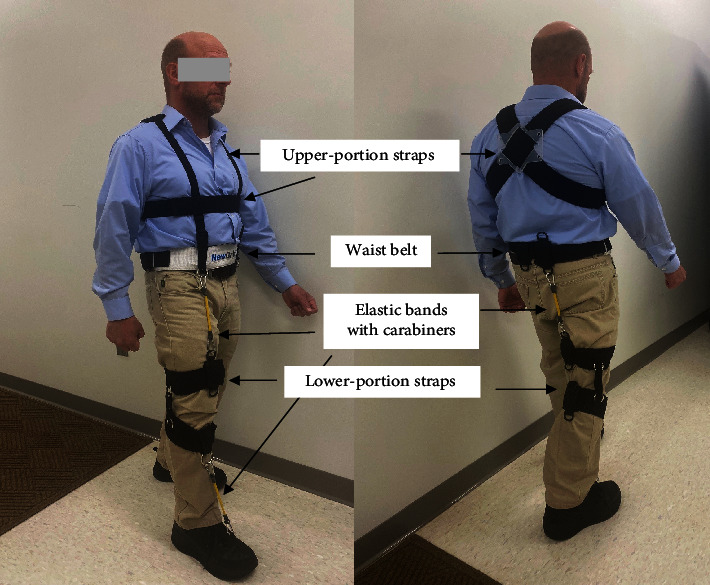
The NewGait rehabilitative device with all components.

The effects of wearing the NewGait rehabilitative device were tested and compared to wearing a regular off-the-shelf posterior leaf spring AFO in an experimental quasiexperimental study. The mechanism of action of the NewGait involved utilizing the properties of elastic bands, as illustrated in Figure 1. The elastic bands were attached with carabiners to the waist belt and stable straps and wrapped around the lower thigh and upper shank, depending on the configuration. Distally, the bands were hooked over the shoelaces. By crossing a single joint, such as the hip or ankle joints, each band brought the distal segments of the lower extremity closer together, aligning joint operation within the normal range of motion necessary for successful completion of specific gait phases or subphases where most gait impairments occur in this population. For individuals with lower extremity paresis, particularly in the swing phase where the foot needs to clear the ground without tripping, dorsiflexion of the ankle and flexion of the hip are essential. Joint approximation was not the sole mechanism of action. The elastic bands came in three different resistance levels (mild, moderate, and strong) and were attached parallel to the line of muscle action, stretching together with the target muscle and then recoiling back, generating an additional contractile force to aid muscle effort.

**Figure 2:**
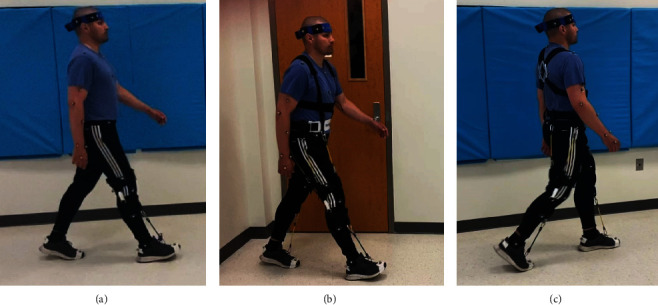
Study participant walking while wearing (a) the NewGait dorsiflexion rehabilitative device, (b) the NewGait 3D hip+dorsiflexion rehabilitative device side view, and (c) the NewGait 3D hip+dorsiflexion rehabilitative device back view. Consent to use the photo for publication has been obtained from the participant.

The NewGait device was used in two different configurations and was composed of several elastic bands attached to stable straps around the lower thigh and waist belt (Figure 2). The first configuration, the NewGait Dorsiflexion (weight = 250 g), helped ankle joint motions of the paretic leg, mostly assisting dorsiflexion and, in some participants, everting the foot. The second configuration, the NewGait 3D hip+dorsiflexion (weight = 360 g), provided assistance to the hip, knee, and ankle joints bilaterally, mostly in the sagittal plane. Based on the participants' presentations, assistance could also be provided in the frontal plane to reinforce the abduction of the hip and the eversion of the foot joints. The second NewGait configuration also included upper-portion straps crossing the shoulders and wrapping around the lower chest. This addition allowed for the erection of the trunk and shoulders, thereby reducing trunk asymmetry and helping with postural correction. The configuration of the bands and straps in both NewGait setups was selected based on the patient's gait impairments and needs.

During a single experimental session, participants walked at their comfortable (normal) speed for a 12 m distance under four different conditions: (1) wearing no assistive device (no assistance); (2) wearing an off-the-shelf posterior leaf spring AFO, either personal or provided by the study investigators (AFO assistance); (3) wearing the NewGait dorsiflexion rehabilitative device (NewGait dorsiflexion assistance); and (4) wearing the NewGait 3D hip+dorsiflexion rehabilitative device, providing full assistance (NewGait full assistance). Participants who wore their own AFO continued to use their personal devices. However, some participants did not regularly use assistive devices despite being prescribed them, with inconvenience being cited as a primary reason. This inconvenience was especially emphasized when transitioning between different daily activities, like walking and sitting. For the purposes of testing, these participants were provided with a plastic AFO of the appropriate size.

Equipping participants with NewGait involves several steps. After the intended set of bands was installed, the participant was asked to walk for 3–5 min with rest breaks to acclimate to the changes the NewGait imposed. Necessary band adjustments were made according to the patient's subjective feelings and observational gait analysis by a physical therapist with more than 30 years of experience in working with neurological patients. A similar adaptation procedure was applied when participants switched to a given AFO, except that no adjustment to the orthosis could be made. The order of the testing conditions was randomized between the participants.

### 2.3. Data Collection and Analysis

The movements of the participants while walking were captured using a Vicon T160 Motion Capture system, which consisted of 12 cameras recording at a rate of 100 Hz. A total of 39 markers were placed on the participants' bodies based on the Plug-in-Gait full body model. Gait events, such as left- or right-foot contacts, were manually identified during the data analysis process. Each gait cycle was defined as the time or distance between two consecutive foot contacts. The collected data were then used to calculate various spatiotemporal and kinematic gait characteristics, as well as the decomposition index.

The spatiotemporal gait characteristics included the gait velocity, step length, step width, foot clearance, and double support time. The kinematic parameters measured in this study included the maximum angular displacements of the hip, knee, and ankle joints in the sagittal plane. To analyze the coordination between different joints, a decomposition index was calculated. This index was based on a method introduced by Bastian et al. in 1996 [13] and was previously validated in a study on individuals with brain injury [14]. In this study, decomposition referred to the pattern of angular motions at three pairs of joints (hip–knee, knee–ankle, and hip–ankle), where the motion at one joint in each pair was paused. The motion was considered paused when the angular velocity dropped below 5% of the baseline velocity. The duration of paused movement was normalized against the duration of the gait cycle to determine the percentage of time when only one joint was in motion. A value of 0% indicated simultaneous motion at both joints, while 100% indicated motion at only one joint. The decomposition index was calculated separately for each pair of joints, specifically for the paretic or more impaired leg.

In addition to the quantitative gait characteristics, upon completion of the gait testing, each participant assessed the comfort level of walking with the NewGait on a 10-point numerical visual analog scale, where 0 represented *very comfortable* and 10 represented *very uncomfortable*.

### 2.4. Statistical Analysis

Despite the relatively small sample size (16 participants), parametric statistical tests were used, as data normality was verified using the Kolmogorov–Smirnov test. Each outcome measure was averaged across three to four repeated strides within each walking trial and then across three consecutive trials for each condition and for each participant. A one-way analysis of variance (ANOVA) with Tukey's honest significant difference (HSD) post hoc test was used to compare averaged means with the factor “walking condition” (no assistance, AFO assistance, NewGait dorsiflexion assistance, and NewGait full assistance). Partial eta squared effect sizes (*η*^2^) were calculated for all parameters and defined as small (*η*^2^ = 0.02), medium (*η*^2^ = 0.13), and large (*η*^2^ = 0.26) effects. A minimum significance level of *p* < 0.05 was set for all the above comparisons. The results of the comfort level assessment were compared between the three conditions (AFO assistance, NewGait dorsiflexion assistance, and NewGait full assistance) using paired *t*-tests, with a significance level of *p* < 0.03 (Bonferroni adjusted).

## 3. Results

### 3.1. Spatiotemporal and Kinematic Gait Characteristics

Table 1 shows the average means and standard deviations of the spatiotemporal and kinematic characteristics for each walking condition. An average gait velocity of 0.65 m/s across participants (range 0.24–1.13 m/s), with more than 20% of the gait cycle spent in double support when walking without orthotic devices, was indicative of moderate to severe gait impairments, defining our participants as limited community ambulators [11]. A one-way ANOVA, applied to the spatiotemporal parameters, showed a difference in only two out of the five characteristics. Wearing an assistive device significantly increased gait velocity (*F*_3,60_ = 36.8, *p* = 0.001, *η*^2^ = 0.36) with the greatest improvements when wearing the NewGait full assistance configuration (post hoc *p* = 0.027), compared to walking with no assistance. Foot clearance also increased with the use of assistive devices (*F*_3,60_ = 16.9, *p* = 0.009, *η*^2^ = 0.16), with no difference between the testing conditions in which assistance was used (post hoc *p* = 0.065). Double support time, step length, and step width also showed a tendency to improve but did not reach significance.

When walking without assistance, the angular motions at the hip, knee, and ankle joints were initially below the minimum range of motion required for comfortable ambulation in healthy individuals. In order to ambulate at an average speed, a minimum of 30° of hip flexion, 60° of knee flexion, 15° of dorsiflexion, and about 20° of plantarflexion are required. The maximum angular displacement in the above joints was much smaller, indicating the presence of gait impairments in the study participants. Wearing orthotic devices helped increase ankle dorsiflexion (*F*_3,60_ = 11.9, *p* = 0.008), with no significant difference between the devices (post hoc *p* = 0.103). However, wearing the AFO significantly reduced plantarflexion ROM (post hoc, *p* = 0.041). No significant differences were found in other comparisons.

### 3.2. Decomposition Index

The decomposition index was defined as the percentage of the gait cycle when motions in a pair of joints lost their synergistic pattern, with one joint moving at a time while the other joint was frozen. An acceptable level of decomposition of the lower extremity joints in healthy individuals falls within a window of 5%–10% when walking at a comfortable speed. In individuals with neurologically induced gait impairments, decomposition may reach or even exceed 20% of the joint motion time [14]. A similar tendency was observed in the present study, whose decomposition exceeded 10%, with the greatest numbers characterizing coordination between the knee and ankle joints (Table 2). ANOVA revealed significant changes in hip–knee decomposition (*F*_3,60_ = 42.5, *p* = 0.001, *η*^2^ = 0.46) when participants wore orthotic devices. Interestingly, wearing an AFO increased the knee-ankle decomposition index (post hoc *p* = 0.017), while wearing the NewGait rehabilitative device, assisting either dorsiflexion or providing full assistance, significantly reduced the index (post hoc *p* = 0.013) compared to walking with no assistance. A similar tendency was observed in coordination involving two other joint pairs. Hip–ankle decomposition increased when wearing an AFO (*p* = 0.022) and remained unchanged when wearing both NewGait devices. Knee–ankle decomposition coordination was reduced by wearing the NewGait dorsiflexion (*F*_3,60_ = 27.5, *p* = 0.078, *η*^2^ = 0.56) compared to walking with no assistance. The coordination was worsened when participants walked with an AFO (post hoc *p* = 0.048).

### 3.3. Comfort Level

After walking with orthotic devices, the participants rated each of them on a 0–10 numerical visual analog scale for pain adapted for the present study, where 0 represented *very comfortable* and 10 represented *uncomfortable*. Participants were also given the option to comment on device comfort in an open-ended format. All three devices were rated as more comfortable than uncomfortable, with scores ranging from 0 to 9. The average score for the AFO device was 4.3 ± 2.9 pts; for the NewGait dorsiflexion device, it was 4.5 ± 2.3 pts; and for the NewGait 3D hip+dorsiflexion, it was 4.4 ± 2.3 pts. A paired *t*-test showed no differences in the participants' perceptions and subjective feelings about the NewGait and AFO devices. Qualitatively, participants noted that donning the NewGait 3D hip+dorsiflexion, providing full bilateral assistance, was not easy for a person with hemiparesis involving the upper extremity, which most of our subjects had, and “required the involvement of a second person.” Participants commented that they “felt very upright and stable when wearing the NewGait 3D hip and dorsiflexion configuration.”

## 4. Discussion

The study results confirmed the effective correction of foot drop using the NewGait rehabilitative devices. Participants wearing the device, configured in two different sets, increased their walking speed and foot clearance, similar to the conditions in which they walked with a regular plastic AFO. In contrast to the AFO, wearing the NewGait did not restrict the plantarflexion range of motion and did not affect interjoint coordination in the paretic leg during walking. Participants reported that the NewGait device is comfortable to wear and does not cause any significant discomfort, similar to wearing a regular AFO. They also mentioned that the device is lightweight and can be used with a variety of footwear without any restrictions.

The increasing gait speed due to wearing the NewGait was anticipated and most likely attributed to the mechanisms of action of the elastic bands. First, the elastic band, crossing a single joint or multiple joints, approximates the distal (to the reference joint) segments of the lower extremity, thereby bringing the joint operation into the normal range of motion for this gait subphase. Specifically, such an approximation increases the dorsiflexion of the ankle joint, which is necessary during the swing phase. This joint approximation approach not only mechanically corrects the segmental length and position but also increases the proximity of the origin and insertion ends of the muscles, which helps reset the sarcomere and muscle length. Normalizing muscle length returns the muscle to its original activation threshold, thereby making this muscle function, at least more effectively, if not back to its original state. This is especially beneficial when a muscle is nonfunctionally elongated due to weakness or increased tone of the muscle antagonist. The approximation mechanism is an effective neuromuscular facilitation technique and is often used in neurorehabilitation in other forms [15–17]. Because an approximation effect was provided by all three assistive devices, both the NewGait(s) and AFO, this explains the similarity of their orthotic correction effects on the gait deviations in the study participants.

The second mechanism of the NewGait device results from the greater mechanical elegance and subsequent nuanced force application compared to the simple rigid plastic arms of the AFO. Whereas the plastic arms of an AFO set the joint in a fixed position or significantly restrict the range of motion, the elastic bands of NewGait allow for more flexibility of joint motions in all orthogonal planes. At the ankle joint, a full range of dorsiflexion and plantarflexion was allowed without affecting the antagonistic action of this muscle pair. Therefore, the stored energy of stretch reflexes is fully utilized during gait [18, 19]. Specifically, for this muscle pair, the active or passive plantarflexion that occurs in the preswing subphase can stretch the dorsiflexors. In response to stretching, when it reaches a threshold necessary to activate the contractile mechanism, the dorsiflexors produce a reflexive muscle contraction, which contributes to the centrally produced voluntary contraction of the same muscle needed to pass the foot through the swing phase without tripping over the ground [20]. Typically, the CNS utilizes muscle stretch reflexes in conjunction with centrally triggered voluntary actions to facilitate quick voluntary muscle contraction and to increase muscle strength when maximal/submaximal, concentric, or eccentric contraction is required [21, 22]. Another very important feature of NewGait for multiplanar joint motions is that the device likely aids synergistic coordination of multiple joints and motion planes by facilitating ankle inversion/eversion, hip abduction/adduction, and rotation, which are necessary for effective ambulation.

Finally, the elastic bands of NewGait resemble the actual skeletal muscle. The bands are attached externally along the actual muscle line of action. When stretched by changes in joint position, these bands generate resistance similar to the contractile response of an actual muscle. Therefore, NewGait improved walking by facilitating the contraction of weak muscles, adding external contraction torques, and simply approximating joints. Although indirectly, the above-discussed features could suggest the positive rehabilitation effect of NewGait in improving gait.

The effects of the NewGait device could also be attributed to the fact that this device did not affect interjoint coordination or range of motion (plantarflexion) more than those already compromised by neurologic injury. A decomposition index was used as an indicator of interjoint coordination, which is essential for all types of multijoint motions, of which gait is a classic representative. Lower index numbers indicate good coordination, in which the paired joints move synergistically and simultaneously. In contrast, higher numbers mean that an individual tends to freeze motion at one joint in favour of other controlled activities [14]. From the first perspective, such a strategy seems to help minimize the accumulated error and save energy. However, it heavily affects movement coordination, making the motion less accurate, flexible, smooth, and efficient. Considering that the human body has hundreds of joints moving simultaneously, the restriction applied to even one joint pair significantly affects the quality of motion performance and eventually leads to an increased risk of injuries and deterioration of motor skills [23]. In this regard, the fact that the NewGait not only did not exacerbate coordination issues but also reduced this sign of incoordination in some joint pairs may be seen as a significant positive effect of the NewGait device. It is currently too early to determine the superiority of the NewGait device over a regular plastic AFO. AFOs still remain the preferred choice for individuals with foot drop. The data were collected during a single experimental session only, and further evidence is needed to validate such claims. However, based on the available data, it is clear that the NewGait has the potential to offer similar, if not superior, improvements for gait.

## 5. Conclusion

In summary, the present study confirmed the usability and immediate positive effects of wearing the NewGait rehabilitative device on gait characterized by foot drop in participants with neurological impairment. This study was a pilot exploration and included a relatively small sample of patients with foot drops that could have affected the results. No difference was found in the perceived comfort level of wearing either the NewGait or AFO. Participants rated both types of devices as comfortable rather than uncomfortable (four out of 10 points). This finding was anticipated, as wearing additional external devices is often perceived as a restriction compared to not wearing any device [24]. However, adding more detailed qualitative assessments to the present study could have revealed a clearer picture of the positively versus negatively perceived features of the NewGait design. This question should be addressed in future studies. Finally, the current study only examined the immediate effects of short-term wearing of the NewGait device. The long-term impact of wearing this device, especially during daily walking and other activities, remains unclear. This was another limitation of the current study, which is expected to be addressed in future clinical trials.

Therefore, the study provided pilot data sufficient for designing future large-scale clinical studies. Specifically, it became clear that walking while wearing the device should take longer to achieve a more pronounced effect. Additionally, whether the device is more effective than other analogous devices can only be determined by comparing different options within the framework of randomized controlled trials. Future clinical trial designs should also incorporate not only spatiotemporal and kinematic gait characteristics but also a more robust set of clinical tests and measures. These should assess the changes induced by NewGait at the impairment, functional limitations, and quality of life levels in individuals with foot drop.

## Figures and Tables

**Table 1:** Participants' spatiotemporal and kinematic gait characteristics.

**Characteristics**	**Testing conditions**			
	**No assistance**	**AFO assistance**	**NewGait dorsiflexion assistance**	**NewGait full assistance**
Gait velocity (m/s)	0.65 ± 0.25	0.71 ± 0.16^∗^	0.72 ± 0.18^∗^	0.77 ± 0.12^∗^^,^^∗∗^
Step length (m)	0.50 ± 0.09	0.52 ± 0.12	0.51 ± 0.10	0.53 ± 0.11
Step width (m)	0.21 ± 0.06	0.19 ± 0.06	0.18 ± 0.05	0.19 ± 0.05
Double support time (% gait cycle)	32.0 ± 11.4	30.6 ± 9.94	33.2 ± 11.9	30.0 ± 8.85
Foot clearance (m)	0.16 ± 0.05	0.18 ± 0.03^∗^	0.18 ± 0.04^∗^	0.19 ± 0.02^∗^
Max hip flexion (°)	26.8 ± 7.8	29.0 ± 10.2	28.5 ± 8.2	27.5 ± 6.9
Max knee flexion (°)	35.7 ± 12.8	37.0 ± 10.6	39.5 ± 5.16	36.2 ± 7.28
Max ankle plantarflexion (°)	24.6 ± 4.21	19.5 ± 4.29^∗^	26.5 ± 7.23	28.0 ± 6.31
Max ankle dorsiflexion (°)	9.6 ± 4.21	14.5 ± 3.29^∗^	14.3 ± 5.2^∗^	12.0 ± 6.88

*Note:* Data are reported as the mean ± SD.

^*^indicates significant differences between walking with no assistance and walking with AFO, NewGait dorsiflexion, or full assistance.

^**^indicates significant differences between walking with AFO assistance and walking with NewGait dorsiflexion or full assistance.

**Table 2:** Decomposition indexes characterizing participants' gait characteristics.

**Characteristics**	**Testing conditions**			
	**No assistance**	**AFO assistance**	**NewGait dorsiflexion assistance**	**NewGait full assistance**
Hip–knee decomposition (%)	16.1 ± 2.81	21.7 ± 2.02^∗^	11.5 ± 6.82^∗^^,^^∗∗^	10.8 ± 5.63^∗^^,^^∗∗^
Hip–ankle decomposition (%)	13.7 ± 1.09	20.2 ± 2.93^∗^	12.2 ± 4.06^∗∗^	14.7 ± 5.32^∗∗^
Knee–ankle decomposition (%)	21.7 ± 4.51	25.8 ± 5.84^∗^	15.1 ± 4.05^∗^^,^^∗∗^	18.4 ± 3.43^∗^^,^^∗∗^

*Note:* Data are reported as the mean ± SD.

^*^indicates significant differences between walking with No assistance and walking with AFO, NewGait dorsiflexion, or full assistance.

^**^indicates significant differences between walking with AFO assistance and walking with NewGait dorsiflexion or full assistance.

## Data Availability

Data associated with the manuscript are available upon request to Ksenia I. Ustinova at ustin1k@cmich.edu.
